# Salutatory

**Published:** 1860-01

**Authors:** 


### EDITORIAL DEPARTMENT.

*Salutatory.*—With the present number commences the twentieth volume of the American Journal of Dental Science. Like all other periodicals which depend upon a special class for support, it has been compelled to struggle long and earnestly, not for profit but for existence. The oldest dental journal in the world, it was brought out at a time when the profession was smaller in numbers and feebler in every respect than it is at present. The line between the charlatan and the scientific practitioner was less sharply drawn, and the whole status of the profession was much below what it is at present. Under these circumstances, such a journal was a rather desperate undertaking, and our readers will not be surprised to learn that for years this publication was a constant source of loss to its proprietors. It did not pay its own expenses, and these had of course to be borne by those who had made up their minds to support the enterprize. It was no small burden but it was cheerfully borne, and year after year, the cash account was balanced by contributions from the pockets of those who were laboring to elevate the standard of the profession by the most effectual of all means, a periodical literature.

It is a source of gratification to be able to inform our readers and subscribers, that these strnggles are now at an end. We commence the new volume under most favorable auspices, with a very gratifying subscription list and a satisfactory state of accounts. The Journal is no longer an experiment, but a fixed institution. Its efforts have been appreciated by the profession generally, and they have rendered it a cordial and efficient support, for which we would tender our thanks did we not feel that it is quite as much their own affair as ours.

We have endeavored, to the extent of our abilities, to make the Journal worthy of the profession to which it is addressed, and we flatter ourselvcs that it has not been the least of the agencies which have effected the change for the better in the intellectual and professional character of dentistry. We say this not in the spirit of boasting, for we are well aware of our own imperfections. One of the greatest drawbacks under which we labor is the difficulty of procuring original articles of merit. There seems to be a strange unwillingness on the part of our ablest men to give to the public the results of their observation and experience. To what cause we must attribute this singular reluctance we are unable to say, but that it exists, the whole profession is as well aware as we.

Nevertheless, under all these disadvantages, we feel that we have presented the profession with many valuable papers, and kept them *au courant* with the scientific progress of the age. We have endeavored, as in a mirror, faithfully to reflect the form and body of the times, with what success it is for our readers to determine. We promise then, not to relax our exertions, but to continue pursuing on towards our ideal of perfection in a journal. We have made arrangements for the coming year which we trust will show themselves in a marked improvement in our original and selected departments. We shall continue to give the latest improvements in science, and in art pertaining to dentistry, and ask the intellectual as well as the pecuniary aid of our friends.

